# Safety, immunogenicity and immune-persistence of a lyophilized human rabies vaccine (Vero cells) under Zagreb and Essen regimens: a randomized, open-label, controlled phase III clinical trial in healthy participants aged 10–60 years in China

**DOI:** 10.3389/fimmu.2024.1444686

**Published:** 2024-11-07

**Authors:** Zhenzhen Liang, Xu Chen, Bo Xing, Xiaosong Hu, Miaomiao Liu, Xinpei Zhang, Yugang Shen, Yan Wang, Yingping Chen, Huakun Lv, Yu Mao

**Affiliations:** ^1^ Department of Immunization Program, Zhejiang Center of Disease Control and Prevention, Hangzhou, Zhejiang, China; ^2^ National Institute of Diagnostics and Vaccine Development in Infectious Diseases, School of Public Health, Xiamen University, Xiamen, Fujian, China; ^3^ Liaoning Chengda Biotechnology Co., Ltd, Shenyang, Liaoning, China; ^4^ Shangyu Center for Disease Control and Prevention, Shaoxing, Zhejiang, China; ^5^ Shengzhou Center of Disease Control and Prevention, Shaoxing, Zhejiang, China

**Keywords:** lyophilized human rabies vaccine (Vero cells), Essen and Zagreb regimens, immunogenicity, safety, non-inferiority (trials)

## Abstract

**Background:**

Rabies continues to be a significant global public health concern, particularly in the Asia region where it is associated with high mortality rate. The administration of effective vaccination is essential in preventing this potentially fatal viral infection. The objective of this study was to evaluate the immunogenicity and safety of two rabies vaccination schedules: the Zagreb (2–1–1) and Essen (1–1–1–1–1) regimens, in a cohort of healthy Chinese individuals aged 10-60 years.

**Methods:**

We conducted a randomized, open-label, controlled, non-inferiority phase 3 trial from July 2021 to November 2022, enrolling a total of 1200 participants. Participants were randomly assigned to receive either the Zagreb or Essen vaccination regimen. The primary outcomes were safety, immunogenicity, and immune persistence. Safety was monitored through adverse event reporting, while immunogenicity was determined by measuring rabies-virus-neutralizing antibody (RVNA) concentrations using the rapid fluorescent focus inhibition test (RFFIT). Immune persistence was evaluated at 3, 6, and 12 months post-vaccination.

**Results:**

The two vaccination regimens exhibited comparable safety records, with mild and transient adverse events predominantly occurring within 0-3 days post-vaccination. The Zagreb regimen demonstrated non-inferiority in terms of seroconversion rates and geometric mean concentrations (GMCs) of antibodies compared to the Essen regimen at both 14 days post-first vaccination and 14 days post-full vaccination. Additionally, both groups displayed nearly 100% seropositivity rate at 3,6, and 12 months. No serious adverse events associated with vaccination were reported.

**Conclusion:**

The findings of this Phase 3 clinical trial provide compelling evidence that the Zagreb regimen is a feasible alternative when compared to the Essen regimen for rabies vaccination, offering a more pragmatic and cost-efficient approach to rabies prevention and control.

**Clinical trial registration:**

http://www.chinadrugtrials.org.cn, identifier CTR20210426.

## 1 Introduction

Rabies is a serious global public health issue. It is a viral disease that primarily affects mammals, including humans. Annually, approximately 35,172 human fatalities (59.6% of global deaths) and around 2.2 million disability adjusted life years (DALYs) are reported in the Asia (1). Rabies virus targets the central nervous system and is mainly transmitted through bites or scratches from infected animals (2, 3). Prevention of disease onset is critical, China takes numerous effective measures to control rabies, such as issuing guidelines for the disposal of rabies exposure and standardizing urban dog breeding (4, 5).

Vaccination with the rabies vaccine is a vital measure for controlling the rabies and protecting human populations from its devastating effects. Different vaccination protocols are used worldwide to intramuscularly administer the rabies vaccine, with the most common being the Essen regimen (1–1–1–1–1) and Zagreb regimen (2–1–1). The Essen regimen prescribes 1 dose on days 0, 3,7,14 and 28, respectively. The Zagreb regimen prescribes 2 doses on day 0, and 1 dose on days 7 and 21, respectively.

The Essen regimen is a gold standard for assessing the immunogenicity and safety of the rabies vaccine; however, its greater cost and lengthy duration often hinder adoption and completion rates—especially in economically disadvantaged regions with a high incidence of rabies exposure. In 2018, the 4-dose Essen regimen (1–1–1–1–0) was recommended by World Health Organization(WHO) which prescribes 1 dose on days 0, 3 and 7 and 1 dose between day 14 – 28. The 4-dose Essen regimen takes a shorter time and less dosage than the 5-dose Essen regimen, which is more affordable to the vaccinees, but it is not approved by National Medical Products Administration (NMPA) yet (6, 7). In 1992, WHO recommended the more cost-effective “2-1-1” Zagreb regimen (8). The data from clinical trial and meta-analyses demonstrates the Zagreb regimen has a good performance in immunogenicity and safety (3, 9, 10). But NMPA did not approve this regimen until 2010, and its implementation and adoption rate still lag behind the Essen regimen.

The study vaccine encapsulated in liquid form was approved the Zagreb regimen (2–1–1) in 2010, which is the earliest rabies vaccine to use the Zagreb vaccination regimen in China (11). In order to improve stability in storage and transportation, the study vaccine is encapsulated in a lyophilized form which was not approved for the Zagreb regimen. We conducted a clinical trial to assess the study vaccine encapsulated in lyophilized form, which concluded in 2019. Under the requirements outlined in “Notification of Approval for Supplementary Drug Application (number:2020B04003)” issued by the NMPA in 2020, we conducted a reassessment of a phase 3 clinical trial employing a randomized, open-label controlled non-inferiority trial design, aiming to evaluate further immunogenicity and safety of lyophilized human rabies vaccine (Vero cell) and employing the Zagreb regimen among healthy individuals aged 10-60 years while exploring immune persistence between these two regimens.

## 2 Materials and methods

### 2.1 Study design and participants

This was a randomized, open-label, controlled, non-inferiority phase 3 trial conducted between July 2021 and November 2022 at the Center for Disease Control and Prevention in Shangyu and Shengzhou County, Zhejiang Province, China (Chinadrugtrials.org identifier: CTR20210426). The study protocol and informed consent form were approved by the Ethics Committee of the Zhejiang Provincial Center for Disease Control and Prevention (approval number: 2021-001-01). Signed informed consent was obtained from participants aged 18-60 years and from participants aged 10-17 years and their guardians before screening, and the trial was conducted in accordance with the Helsinki Declaration, Good Clinical Practice, and Chinese regulatory requirements.

The study planned to enroll 1200 healthy Chinese volunteers aged 10-60 years. The eligibility of participants was assessed through medical history inquiry and physical examination by the investigators. The exclusion criteria included: any previous rabies vaccination; any bites or scratches from dogs or other mammals within past 6 months; receipt of immunoglobulins, blood or blood-derived products within the past 3 months; any specified comorbidities that may influence the immune response of vaccination or lead to severe adverse events (AEs), and any prior administration of the vaccines, investigational products, within a defined period.

### 2.2 Study vaccine

The study vaccine (Vero cell) is a freeze-dried vaccine developed by Liaoning Chengda Biotechnology Co., Ltd. The vaccine contains inactivated PV strain virus, which is a serum type I rabies virus originating from the rabies virus strain “L. Pasteur 2061”. A lot certificate was provided by NMPA to confirm its eligibility. All vaccines in this study came from the marketed batch and were not specifically manufactured for the clinical trial (batch number: 202007268). Each dose of vaccine was accompanied by a 0.5 ml vial of sterile water for injection, to be used for reconstitution.

### 2.3 Vaccination regimens

In the Zagreb groups, two doses of vaccines were intramuscularly administered into the deltoid muscle of upper arm on day 0, and 1dose on day 7 and 21, respectively. In the Essen groups, each dose of vaccine was given on days 0, 3, 7, 14, and 28. Subjects were randomized in a 1:1 ratio to receive the Zagreb regimen or the Essen regimen.

### 2.4 Safety assessment

Safety data were documented by diary cards. Information on both requested and spontaneous injection site and systemic reactions was gathered, including immediate reactions occurring within 30 minutes of vaccination. Safety data were collected after each vaccination, as well as up to 30 days following the complete vaccination course (requested AEs were collected during Days 0-7). The solicited injection site AEs included pain, redness, swelling, induration, rash and pruritus, while solicited systemic AEs included fever, headache, dizziness, fatigue, diarrhea, abdominal pain, nausea, vomiting, muscle ache, joint pain, and acute allergic reaction. The investigator was responsible for determining the relationship of the study treatment and AEs, as well as assessing the severity of unsolicited AEs.

Adverse reactions (ARs) were defined as AEs associated with the vaccine and were coded using the Medical Dictionary for Regulatory Activities (MedDRA). Serious adverse events (SAEs) for all participants were documented up to 6 months following after the final injection.

### 2.5 Immunogenicity assessment

For immunogenicity assessments, blood samples (approximately 3-4 mL) were collected prior to vaccination (D0), D14, D35(Zagreb)/42(Essen), month 3, month 6 and month12 (only the first 100 participants in each group were assessed at month 12) after the final injection. Rabies-virus-neutralizing antibody (RVNA) concentration levels were determined by means of a Rapid Fluorescent Focus Inhibition Test (RFFIT) performed at the National Institutes for Food and Drug Control (NIFDC, Beijing, China). The positive seroconversion rate was defined as the percentage of the participants with RVNA < 0.5 IU/mL before vaccination and with RVNA ≥ 0.5 IU/mL after vaccination.

### 2.6 Sample size calculation

Sample size was estimated based on the non-inferiority of seroconversion rate and the GMC at 14 days after the first vaccination with the Zagreb or Essen regimens. We assumed a seroconversion rate of ≥ 95% at 14 days after the first vaccination in vaccine-naïve individuals, with a non-inferiority margin of −5%. Assuming a one-sided α of 0.025, the sample size for the primary vaccination phase with the Zagreb or Essen regimens would need to be 483 to achieve a power of 90%. For the GMC, we assumed a non-inferiority margin of -0.17609 (transforming from 2/3 by log10), with the one-sided α set to 0.025. Thus, the sample size for the Zagreb or Essen regimens needed to be 366 to achieve a power of 92.5%. Finally, considering the 20% drop-out rate and the seropositive rate before vaccination, the overall sample size was set at 1,200 (600 in each of the two groups).

### 2.7 Statistical analysis

Safety assessments were performed on safety set (SS) comprising all participants who received at least one dose of the study vaccine. Immunogenicity assessments at 14 days post-first vaccination, 14 days post-full vaccination (D35/42), and the immune-persistence evaluations were performed on the full analysis set (FAS), per-protocol set (PPS), and immune-persistence set (IPS) correspondingly. The FAS included participants who received the first dose of the vaccine, completed pre-vaccination blood sampling, and had valid antibody levels. The PPS included the participants who satisfied the inclusion/exclusion criteria, completed the full vaccination course, underwent blood sampling on D14 and D35/42, and had valid antibody levels among those who were seronegative prior to vaccination. The IPS included all participants who completed the full primary immunization course and underwent immune-persistence blood collection 3 months later with valid antibody levels.

All statistical analyses were performed using SAS statistical software, version 9.4 or higher. The geometric mean concentrations (GMCs) of RVNA and associated 2-sided 95% confidence intervals (CIs) were calculated by exponentiating the least square means and the lower and upper limits of the 95% CIs of the log transformed titers for each regimen. The ratio of GMCs for each age cohort at day 14 between the Zagreb and Essen regimens was computed by a 2-way Analysis of Variance (ANOVA) adjusting for factors of regimen and age subset.

The immunogenicity after vaccination was assessed by the seroconversion rate and the GMCs of antibodies on D14 and D35/42 for participants. The persistence of immunity post-vaccination was assessed by the positive antibody rate and the GMCs of antibodies at 3 months, 6 months, and 12 months post-full vaccination. Categorical data were characterized by the frequency of cases and the percentage representation, and comparisons between groups were made using the unpaired t-test, chi-square test and Fisher’s exact probability method.

The safety analysis was assessed by the incidence and severity of systemic and local AEs and SAEs of two regimen groups. Statistical analysis was performed by an independent statistician. All tests were one-sided, with α set at 0.025.

## 3 Results

### 3.1 Participants

**Figure 1:**
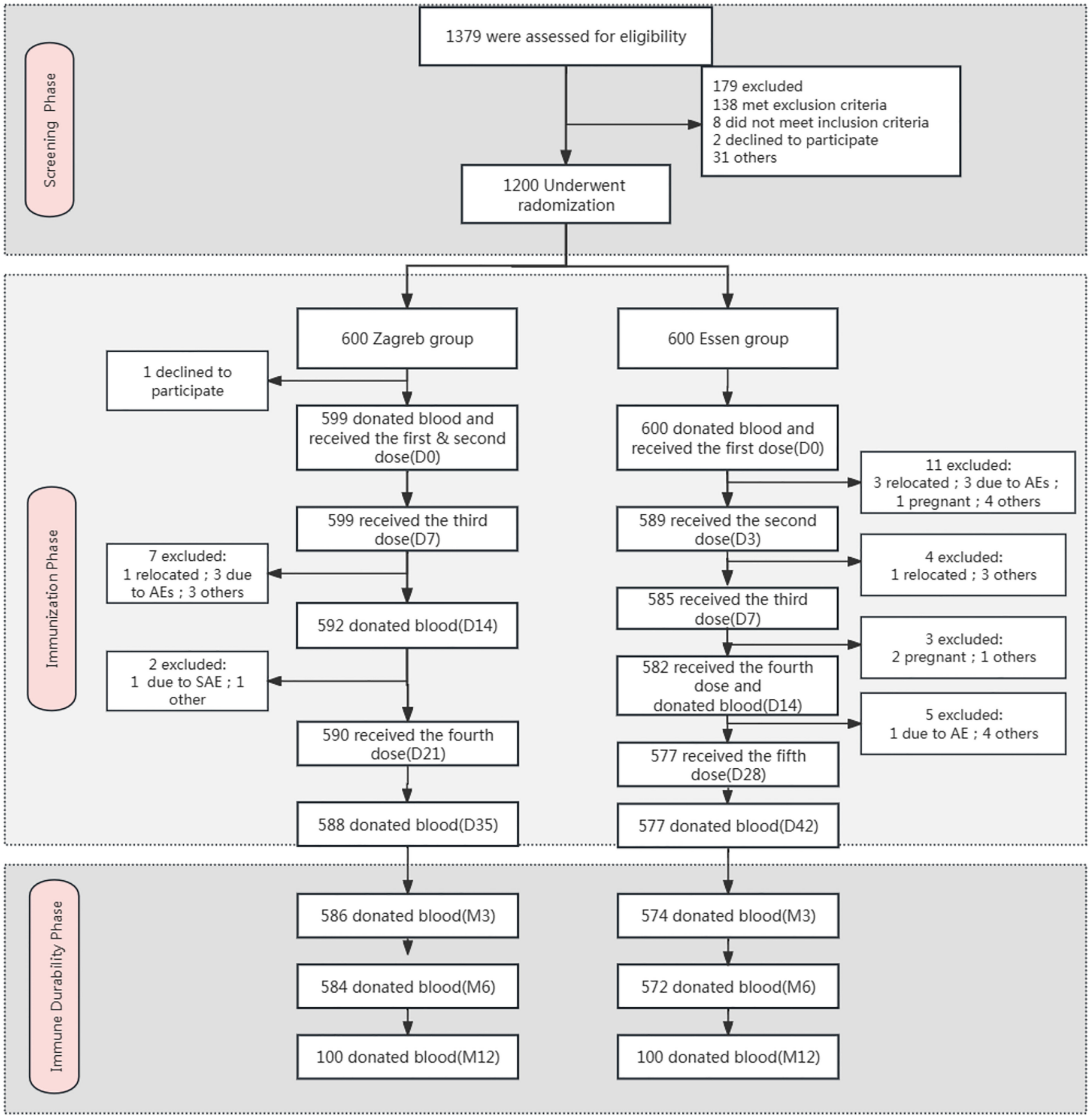
Flow diagram of the trial. *Other: the subject went out due to reasons such as academic studies, employment, family commitments, and personal preferences.

A total of 1379 subjects were screened, of which 179 failed. All 1200 enrolled participants were randomly assigned to the Zagreb group (n=600) or Essen group (n=600). Details are presented in **Figure 1**. The average age of the participants was 35.8 years. Both groups were comparable in terms of mean age, sex, ethnicity, height, and weight, as shown in **Table 1**.

**Table 1:** Baseline characteristics of the study participants in safety set.

Characteristics	Zagreb group (N=599)	Essen group (N=600)	*P value*
Age(years), mean ± SD	35.80 ± 13.60	35.70 ± 13.50	0.892
Male [n (%)]	273 (45.58)	268 (44.67)	0.752
Ethnic Han [n (%)]	594 (99.17)	595 (99.17)	1.000
Height (m), mean ± SD	162.50 ± 8.10	162.70 ± 8.30	0.676
Weight (kg), mean ± SD	63.02 ± 12.37	62.84 ± 12.69	0.805
Major medical history [n (%)]	11 (1.84)	14 (2.33)	0.547

For the Safety Set (SS) and Full Analysis Set (FAS), 1199 subjects were included, comprising 599 (99.83%) from the Zagreb group and 600 (100%) from the Essen group. The Per-Protocol Set (PPS) included 1015 subjects, with 519 (86.6%) from the Zagreb group and 496 (82.6%) from the Essen group. Additionally, the Immune Persistence Set (IPS) included 1160 subjects, 586 (97.7%) from the Zagreb group and 574 (95.7%) from the Essen group.

### 3.2 Safety

The overall incidence of AEs within 30 days after vaccination was 50.71% (608/1199). The incidence of ARs was 45.41% for the Zagreb group and 42.50% for the Essen group, respectively. No significant differences were found in the incidence of ARs between the Zagreb and Essen groups within 30 minutes, 0-3 days and 0-7 days after vaccination. The ARs were primarily observed within 3 days after vaccination, with an incidence of 43.91% for the Zagreb group and 41.83% for the Essen group. The incidence of ARs after the first dose was higher in Zagreb group than in the Essen group (39.57% and 29.50%) (**Table 2**). The difference was mainly attributed to pain at the injection site (33.89% and 22.67%).

**Table 2:** The occurrence of adverse reactions in immune stage.

Analysis Item	Zagreb group (N=599)		Essen group (N=600)		*P value*
	n	(%)	n	(%)	
**Total Adverse Events**	306	51.09	302	50.33	0.817
30min	36	6.01	40	6.67	0.722
D0-D3	277	46.24	275	45.83	0.908
D0-D7	295	49.25	283	47.17	0.488
D8-D30	42	7.01	35	5.83	0.413
Dose 1	254	42.40	190	31.67	0.001
Dose 2	/	/	124	21.05	/
Dose 3	125	21.11	95	16.24	0.036
Dose 4	83	14.07	77	13.23	0.734
Dose 5	/	/	68	11.79	/
**Adverse Reaction**	272	45.41	255	42.50	0.323
30min	36	6.01	40	6.67	0.722
D0-D3	263	43.91	251	41.83	0.484
D0-D7	272	45.41	255	42.50	0.323
D8-D30	0	0.00	0	0.00	1.000
Dose 1	237	39.57	177	29.50	0.001
Dose 2	/	/	115	19.52	/
Dose 3	100	16.89	77	13.16	0.087
Dose 4	52	8.81	58	9.97	0.548
Dose 5	/	/	37	6.41	/

The incidence of local reactions was 38.56% for the Zagreb group and 34.33% for the Essen group, and the incidence of systemic reactions was 21.37% for the Zagreb group and 20.17% for the Essen group. No significant differences were observed in the incidence rates of AEs and ARs (both local and systemic) between the two groups. The most common AR reported in both groups was pain at the injection site (37.06% for the Zagreb group and 31.67% for the Essen group), followed by weakness (10.02% and 8.33%) and fever (6.68% and 6.83%) (**Tables 2**, **3**).

**Table 3:** Severity of adverse reactions reported within 30 days after vaccine (SS).

Adverse Events	Zagreb group (N=599)		Essen group (N=600)		*P value*
	n	(%)	n	(%)	
**Injection site (local)**	231	38.56	206	34.33	0.134
Pain	222	37.06	190	31.67	0.052
Itching	9	1.50	17	2.83	0.164
Swelling	10	1.67	15	2.50	0.419
Erythema	8	1.34	13	2.17	0.379
Induration	4	0.67	4	0.67	1.000
Rash	1	0.17	0	0.00	0.500
Other	4	0.67	11	1.83	0.116
**Non-injection site (systemic)**	128	21.37	121	20.17	0.619
Weakness	60	10.02	50	8.33	0.319
Fever	40	6.68	41	6.83	1.000
Dizziness	36	6.01	33	5.50	0.712
Headache	30	5.01	25	4.17	0.494
Diarrhea	20	3.34	11	1.83	0.105
Abdominal pain	16	2.67	9	1.50	0.164
Vomiting	3	0.50	8	1.33	0.224
Myalgia (muscle pain)	12	2.00	10	1.67	0.675
Arthralgia (joint pain)	10	1.67	4	0.67	0.116
Hypersensitivity reaction	2	0.33	3	0.50	1.000
Other	4	0.67	4	0.67	1.000

**Figure 2:**
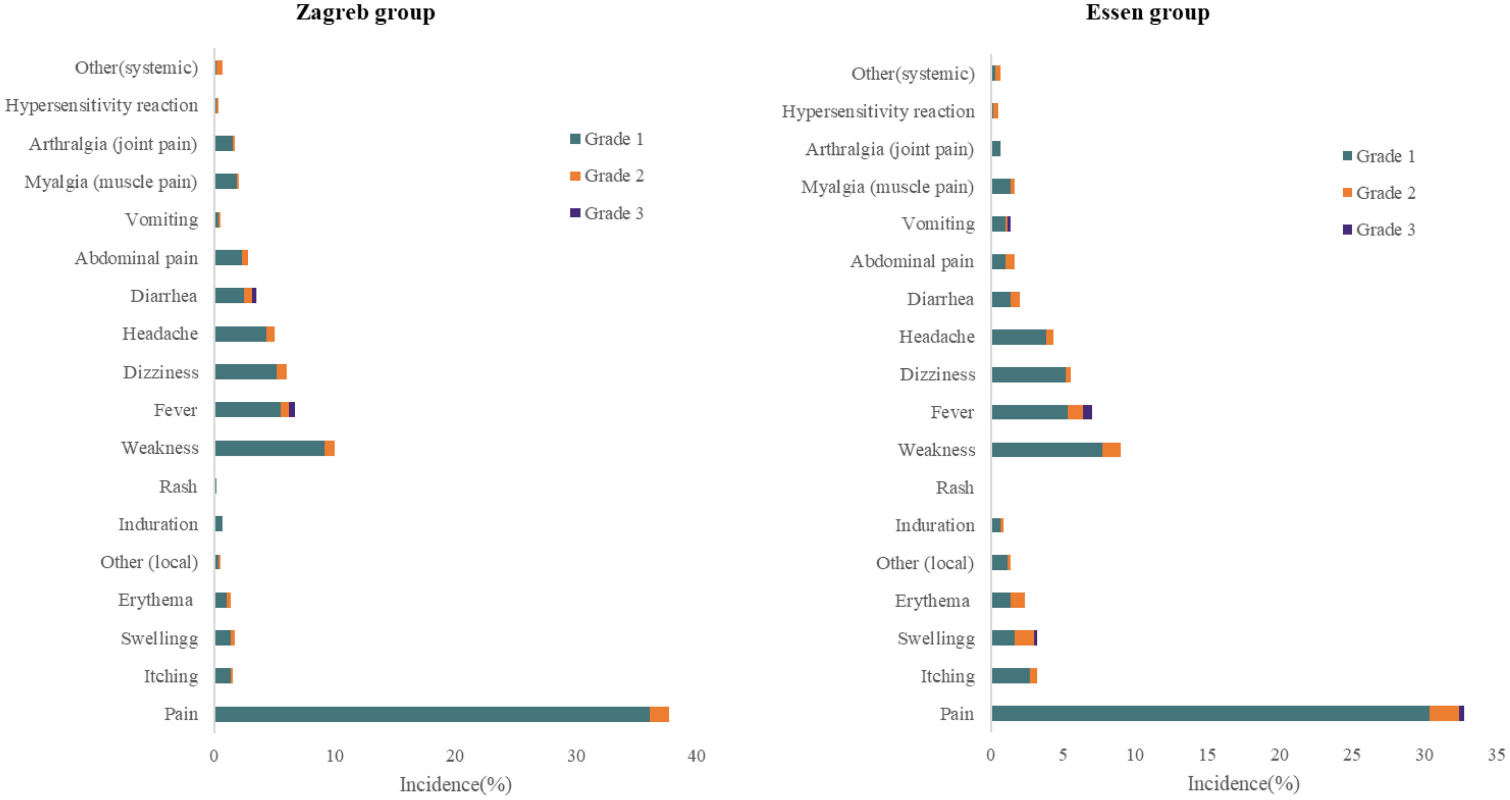
Incidence of solicited adverse reactions reported within 30 days after (SS).

Most of ARs were Grade 1, with the incidence of 44.41% in the Zagreb group and 41.17% in the Essen group, respectively. The incidence of the ARs of Grade 2 was 5.51% for the Zagreb group and 6.67% for the Essen group, and Grade 3 was 0.83% and 1.17% respectively. The most common ARs of Grade 3 was fever. There were no significant differences in the incidence of ARs across different severity levels between the two groups. No ARs of Grade 4 or higher were observed in either group (**Figure 2**).

During the primary vaccination phase, a total of 13 cases (16 episodes) of SAEs were observed, with incidence of 1.00% for the Zagreb group and 1.71% for the Essen group. But all reported SAEs were not related to the vaccination.

### 3.3 Immunogenicity

A total of 120 participants (10.01%) tested positive for antibodies prior to vaccination, with 59 (9.85%) in the Zagreb group and 61 (10.17%) in the Essen group, indicating no statistically significant difference.

In the seronegative population at baseline, the positive seroconversion rate was 100% at 14 and 35/42 days following the first dose in both groups. The lower bounds of the 95% CIs for the difference between the two groups exceeded -5%, and the lower bounds of the 95% CIs for the ratio of GMCs of antibodies between Zagreb and Essen groups were all above 0.67, indicating non-inferiority of Zagreb protocol’s antibody positive seroconversion rate and GMC compared to those of Essen protocol (**Table 4**).

**Table 4:** Comparison of seropositive seroconversion rates (%) and antibody GMC (IU/mL) of participants in different groups after rabies vaccination.

Blood Collection Time	FAS			PPS		
	Zagreb group (N=599)	Essen group (N=600)	*P value*	Zagreb group (N=519)	Essen group (N=496)	*P value*
Pre-Vaccination (D0)						
Positivity Rate	59 (9.85%)	61 (10.17%)	0.855	/	/	/
GMC	0.13 (0.12, 0.14)	0.13 (0.12, 0.14)	0.823	0.10 (0.10, 0.10)	0.10 (0.10, 0.11)	1.000
14 days after the first dose (D14)						
Seropositive seroconversion rate	592 (98.83%)	582 (97.00%)	0.027	519 (100%)	496 (100%)	1.000
GMC	63.75 (58.49, 69.48)	60.91 (54.69, 67.84)	0.517	65.44 (61.33, 69.81)	70.87 (66.33, 75.72)	0.091*
14 days after full vaccination (D35/D42)						
Seropositive seroconversion rate	592 (100%)	577 (100%)	1.000	519 (100%)	496 (100%)	1.000
GMC	37.04 (34.08, 40.26)	32.33 (29.24, 35.75)	0.041	36.83 (34.50, 39.31)	35.80 (33.49, 38.26)	0.551

*Covariance analysis result.

At 14 days after the first dose, the adjusted GMCs of antibodies (95% CI) for the seronegative population prior to vaccination were 65.44 IU/mL (61.33 IU/mL, 69.81 IU/mL) for the Zagreb group and 70.87 IU/mL (66.33 IU/mL, 75.72 IU/mL) for the Essen group. The ratio of adjusted antibody GMCs (Zagreb/Essen) was 0.92 (0.84, 1.01), with the lower bound of the 95% confidence interval exceeding 0.67, indicating non-inferiority of the Zagreb group’s antibody GMC at this time point (**Table 4** for details).

At 14 days after full vaccination, the GMCs of antibodies (95% CI) for the seronegative population were 36.83 IU/mL (34.50 IU/mL, 39.31 IU/mL) for the Zagreb group and 35.80 IU/mL (33.49 IU/mL, 38.26 IU/mL) for the Essen group. The ratio of antibody GMCs (Zagreb/Essen) was calculated to be 1.03 (0.94, 1.13), with the lower bound of the 95% confidence interval exceeding 0.67, indicating a lack of significant difference between groups (*P*=0.5510).

**Figure 3:**
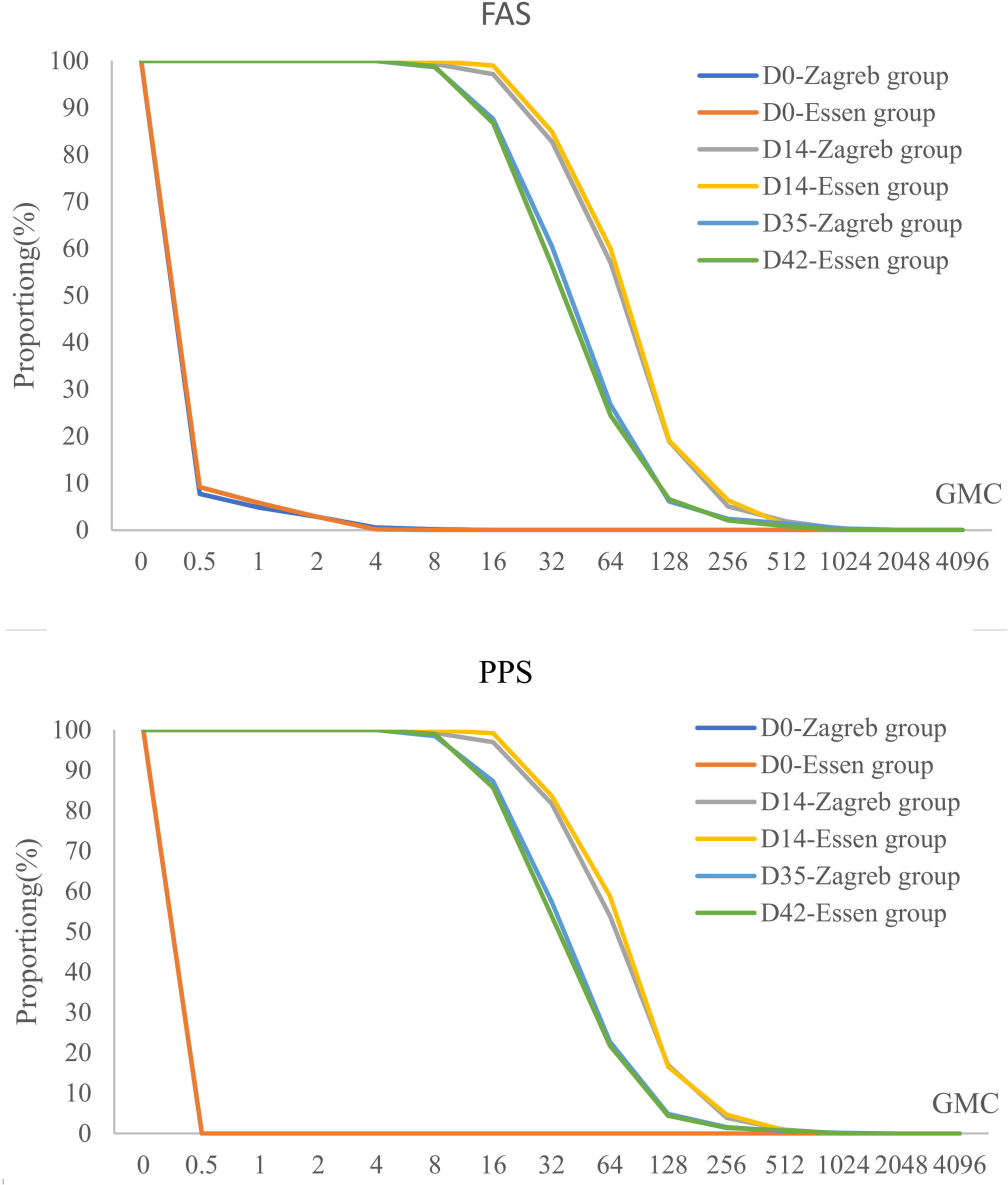
Pre- and post-immunization reverse cumulative distribution in different vaccination groups after rabies vaccination (FAS/PPS).

The Reverse Cumulative Distribution Plot visually depicts the distribution of antibody levels pre- and post-immunization for participants in both experimental groups (**Figure 3**), providing a comprehensive representation of the data.

### 3.4 Immune-persistence

**Figure 4:**
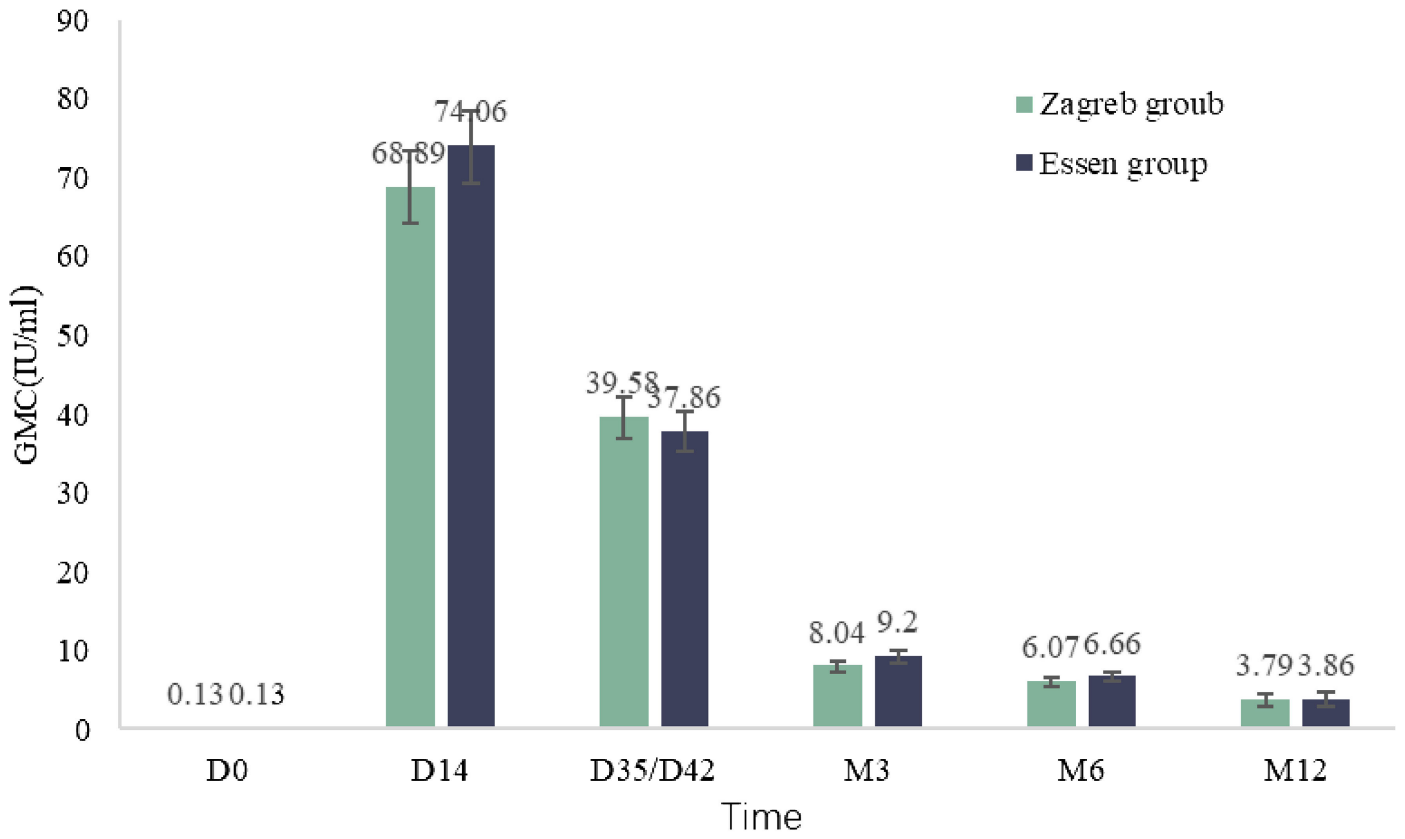
Antibody geometric mean concentrations (GMC) at various post-vaccination observation time points in subjects (IPS).

Both groups showed a 100.00% seropositivity rate at month3 and month 6. At month 12, all participants in the Zagreb group except one, who had seroconverted with a GMC of 0.4 IU/ml, had positive antibodies (positivity rate of 99.0%). The GMC of antibodies decreased significantly at 3 months after the first dose with the GMCs of 8.04 IU/mL (7.37 IU/mL, 8.77 IU/mL) for Zagreb group and 9.20 IU/mL (8.49 IU/mL, 9.98 IU/mL) for Essen group, and gradually decreased at 6 months and 12 months with the GMCs of 3.79 IU/mL (3.02 IU/mL, 4.75 IU/mL) for Zagreb group and 3.86 IU/mL (3.10 IU/mL, 4.82 IU/mL) for Essen group at month 12. There was no significant difference in antibody GMC between the Zagreb and Essen regimen groups at each visit time point (**Figure 4**).

## 4 Discussion

This study aimed to assess the immunogenicity and safety profiles of two distinct rabies vaccination schedules: Zagreb (2–1–1) and Essen (1–1–1–1–1). The findings indicated that the incidence of AEs and ARs between the two groups was not significant, most AEs were mild. In terms of immunogenicity, the Zagreb regimen was non-inferiority to the Essen regimen in terms of seroconversion rates and GMCs of antibodies at 14 days post-first vaccination and 14 days post-full vaccination. Furthermore, the antibody positivity rates at 3, 6, and 12 months after full vaccination were nearly 100%, providing strong evidence for the long-term protective efficacy of the vaccine.

The incidence of AEs between the Zagreb and Essen groups showed numerical differences but were not statistically significant. This may be related to the simultaneous administration of the first dose in both arms on day 0 in the Zagreb group, which was consistent with previous study (12–14). Local pain, systemic weakness, and fever were common ARs, all of them were mild to moderate and transient, aligning with common responses to rabies vaccine administration (15–17). Most AEs occurred after the early doses, particularly the first dose, and the incidence of AEs following each dose decreased with the increase of the number of doses, indicating no dose-related increase in adverse effects from the vaccine (15). No vaccination-related SAEs were observed, further confirming the safety of both regimens.

The Zagreb regimen induced a similar immune response in the short term compared to the traditional Essen regimen. After vaccination, antibody levels in all subjects met the requirements set by the WHO and the Chinese Pharmacopoeia, which stipulate that the potency of human rabies vaccines should be > 4.0 IU/mL and not less than 2.5 IU/mL (9, 18). In this study, serum antibody levels peaked on day 14 (63.15-86.61 IU/mL), significantly exceeding the effective protective level (RVNA ≥ 0.5 IU/mL), crucial for rapidly generating a sufficient antibody to prevent rabies. This finding is consistent with previous research, with antibody values higher than those reported in other studies (5, 19–21), likely due to differences in vaccine production technology and the demographic characteristics of the study population. Compared to 14 days post-first dose, the GMC of antibodies declined 14 days post-full vaccination, with a noticeable decrease starting three months post-vaccination, which showed no inter-group differences between the Zagreb and Essen regimens. The seropositive rate among subjects was nearly 100% at 3, 6, and 12 months post-full vaccination, which was observed in other clinical trials of rabies vaccines (22–24). Among the study population, only one case had an antibody level below 0.5 IU/mL at month 12. This seronegative subject was a 55-year-old female with no underlying diseases, whose antibody GMC at various time points post-vaccination were 0.2 IU/mL, 15.9 IU/mL, 21.2 IU/mL, 2.3 IU/mL, 0.7 IU/mL, and 0.4 IU/mL, which may be related to age-related immunosenescence and the decline in immune function (25).

The study’s findings indicated that the Zagreb regimen had higher compliance than the Essen regimen (17). The Zagreb schedule, by shortening the vaccination period from 28 to 21 days, offers a more practical vaccination regimen with fewer doses and reduced costs, which makes it a promising candidate for broader implementation (18, 26).

In conclusion, the Phase 3 clinical trial substantiates that the Zagreb regimen is not inferior in efficacy to the Essen regimen, with an added advantage of a superior cost-effectiveness ratio. This research provides a robust scientific foundation for the refinement of rabies vaccination protocols, thereby bolstering worldwide initiatives for rabies prevention and control.

However, there is another vaccination regimen named 4- dose Essen regimen which prescribes 1 dose on days 0, 3 and 7 and 1 dose between day 14-28 (27). Apparently, the 4-dose Essen regimen takes a shorter time and less dosage than the 5-dose Essen regimen, which is more affordable to the vaccines, it has not yet been approved by the NMPA. But during the study design period, we considered the 4-dose Essen regimen, and designed a clinical trial to assess its immunogenicity and safety, but the results have not been reported yet (19, 28).

## 5 Strengths and limitations

This study represents a large-scale, phase 3 clinical trial employing a randomized controlled design with rigorous follow-up. Participants were carefully selected according to strict criteria, ensuring the reliability and validity of the findings. The limitations of this study include the inability to track immunogenicity over an extended period and the exclusion of young children and the elderly from the research population.

## Data Availability

The original contributions presented in the study are included in the article/**Supplementary Material**. Further inquiries can be directed to the corresponding authors.
