# Supplementary material for: Safety, immunogenicity and immune-persistence of a lyophilized human rabies vaccine (Vero cells) under Zagreb and Essen regimens: a randomized, open-label, controlled phase III clinical trial in healthy participants aged 10–60 years in China
Source: Front Immunol. 2024 Nov 7;15:1444686. doi: 10.3389/fimmu.2024.1444686 (PMC11578971; doi:10.3389/fimmu.2024.1444686)
Supplement: Supplementary file 1 [file DataSheet1.docx]

Supplementary Material

# Supplementary Methods

**Inclusion and Exclusion Criteria**

**Inclusion Criteria (marked with * indicates that if not met, a re-visit can be conducted):**

1. Age between 10 to 60 years old, with valid identification documents;
2. Volunteers who agree to participate in the study and have signed the Informed Consent Form (for subjects aged 10 to 17, the Informed Consent Form for Minors must be signed, and their legal guardian must also sign the Informed Consent Form);
3. Subjects or their legal guardians are capable of understanding the study procedures (non-illiterate) and can attend all scheduled follow-ups;
4. Axillary temperature on the day of enrollment is less than 37.3°C (for those over 14 years old), and less than 37.5°C (for those 14 years old or younger).*

**Exclusion Criteria for the first dose (marked with * indicates that if met, a re-visit can be conducted):**

1. History of rabies vaccine immunization or use of rabies virus passive immunization preparations;
2. History of bites or scratches from dogs or other mammals (with skin damage) within 6 months prior to the first vaccination;
3. Received blood or blood-related products within 3 months prior to the first vaccination;
4. Used other investigational or unregistered products (drugs or vaccines) within 3 months prior to the first vaccination, or plans to participate in other clinical studies after enrollment in this clinical study (except for emergency use of new coronavirus vaccines that have not been officially approved for market, but the vaccination details must be recorded in detail in the concomitant medication section);
5. Received immunosuppressive therapy within 3 months prior to the first dose (such as long-term systemic corticosteroids for ≥14 days, at a dose of ≥2mg/kg/day or ≥20mg/day of prednisone or equivalent);
6. Vaccinated with any vaccine within 14 days prior to the first vaccination;*
7. Suffered from acute febrile illness (body temperature >38.5°C) and acute phase of infectious diseases, or used antipyretic analgesics or antihistamines within 3 days before vaccination;*
8. History of severe allergies requiring medical intervention, such as anaphylactic shock, allergic laryngeal edema, allergic purpura, thrombocytopenic purpura, local allergic necrotic reaction (Arthus reaction), severe urticaria, etc., or known allergies to any component of the test vaccine (human serum albumin, disodium hydrogen phosphate, sodium dihydrogen phosphate, sodium chloride, dextran 40);
9. Suffer from congenital or acquired immune deficiency or other autoimmune diseases;
10. Suffer from congenital heart disease, developmental disorders, severe liver and kidney diseases, severe diabetes (with complications), malignant tumors, various acute diseases, or acute exacerbation of chronic underlying diseases;
11. Currently have or have a history of convulsions, epilepsy, and other neurological diseases, as well as mental illnesses;
12. Have contraindications to intramuscular injection, such as bleeding disorders, coagulation disorders;
13. Systolic blood pressure ≥140mmHg or diastolic blood pressure ≥90mmHg for those aged 18 and above, systolic blood pressure >120mmHg or diastolic blood pressure >80mmHg for those aged 10 to 17, regardless of medication use;
14. Pregnant women with a positive urine pregnancy test, or during pregnancy or lactation, or planning to conceive during the study period (applicable to women from menarche to menopause);
15. Any condition deemed by the researcher as potentially affecting the assessment of the tria

# Supplementary Tables

Table S1. Incidence of solicited adverse reactions reported within 30 days

| **Severity** | **Zagreb group** | | **Essen group** | | ***P value*** |
| --- | --- | --- | --- | --- | --- |
|  | **(N=599)** | | **(N=600)** | |  |
|  | **n** | **(%)** | **n** | **(%)** |  |
| **Injection site (local)** | 231 | 38.56 | 206 | 34.33 | 0.1338 |
| Grade 1 | 223 | 37.23 | 200 | 33.33 | 0.1648 |
| Grade 2 | 15 | 2.5 | 17 | 2.83 | 0.8581 |
| Grade 3 | 0 | 0 | 3 | 0.5 | 0.2494 |
| Pain | 222 | 37.06 | 190 | 31.67 | 0.0518 |
| Grade 1 | 216 | 36.06 | 182 | 30.33 | 0.0372 |
| Grade 2 | 10 | 1.67 | 12 | 2 | 0.8302 |
| Grade 3 | 0 | 0 | 2 | 0.33 | 0.4996 |
| Itching | 9 | 1.5 | 17 | 2.83 | 0.164 |
| Grade 1 | 8 | 1.34 | 16 | 2.67 | 0.1475 |
| Grade 2 | 1 | 0.17 | 3 | 0.5 | 0.6244 |
| Grade 3 | 0 | 0 | 0 | 0 | 1 |
| Swellingg | 10 | 1.67 | 15 | 2.5 | 0.4194 |
| Grade 1 | 8 | 1.34 | 10 | 1.67 | 0.8131 |
| Grade 2 | 2 | 0.33 | 8 | 1.33 | 0.1079 |
| Grade 3 | 0 | 0 | 1 | 0.17 | 1 |
| Erythema | 8 | 1.34 | 13 | 2.17 | 0.3791 |
| Grade 1 | 6 | 1 | 8 | 1.33 | 0.7893 |
| Grade 2 | 2 | 0.33 | 6 | 1 | 0.2874 |
| Grade 3 | 0 | 0 | 0 | 0 | 1 |
| Other | 4 | 0.5 | 11 | 1.17 | 0.116 |
| Grade 1 | 3 | 0.33 | 10 | 1.17 | 0.091 |
| Grade 2 | 1 | 0.17 | 1 | 0.17 | 1 |
| Grade 3 | 0 | 0 | 0 | 0 | 1 |
| Induration | 4 | 0.67 | 4 | 0.67 | 1 |
| Grade 1 | 4 | 0.67 | 4 | 0.67 | 1 |
| Grade 2 | 0 | 0 | 1 | 0.17 | 1 |
| Grade 3 | 0 | 0 | 0 | 0 | 1 |
| Rash | 1 | 0.17 | 0 | 0 | 0.4996 |
| Grade 1 | 1 | 0.17 | 0 | 0 | 0.4996 |
| Grade 2 | 0 | 0 | 0 | 0 | 1 |
| Grade 3 | 0 | 0 | 0 | 0 | 1 |
| **Non-injection site (systemic)** | 128 | 21.37 | 121 | 20.17 | 0.6188 |
| Grade 1 | 122 | 20.37 | 109 | 18.17 | 0.342 |
| Grade 2 | 22 | 3.67 | 26 | 4.33 | 0.659 |
| Grade 3 | 5 | 0.83 | 5 | 0.83 | 1 |
| Weakness | 60 | 10.02 | 50 | 8.33 | 0.3191 |
| Grade 1 | 55 | 9.18 | 46 | 7.67 | 0.3516 |
| Grade 2 | 5 | 0.83 | 8 | 1.33 | 0.5789 |
| Grade 3 | 0 | 0 | 0 | 0 | 1 |
| Fever | 40 | 6.68 | 41 | 6.83 | 1 |
| Grade 1 | 33 | 5.51 | 32 | 5.33 | 0.8994 |
| Grade 2 | 4 | 0.67 | 6 | 1 | 0.7529 |
| Grade 3 | 3 | 0.5 | 4 | 0.67 | 1 |
| Dizziness | 36 | 6.01 | 33 | 5.5 | 0.712 |
| Grade 1 | 31 | 5.18 | 31 | 5.17 | 1 |
| Grade 2 | 5 | 0.83 | 2 | 0.33 | 0.2874 |
| Grade 3 | 0 | 0 | 0 | 0 | 1 |
| Headache | 30 | 5.01 | 25 | 4.17 | 0.4941 |
| Grade 1 | 26 | 4.34 | 23 | 3.83 | 0.6651 |
| Grade 2 | 4 | 0.67 | 3 | 0.5 | 0.7256 |
| Grade 3 | 0 | 0 | 0 | 0 | 1 |
| Diarrhea | 20 | 3.34 | 11 | 1.83 | 0.1054 |
| Grade 1 | 15 | 2.5 | 8 | 1.33 | 0.1475 |
| Grade 2 | 4 | 0.67 | 4 | 0.67 | 1 |
| Grade 3 | 2 | 0.33 | 0 | 0 | 0.2494 |
| Abdominal pain | 16 | 2.67 | 9 | 1.5 | 0.164 |
| Grade 1 | 14 | 2.34 | 6 | 1 | 0.0758 |
| Grade 2 | 3 | 0.5 | 4 | 0.67 | 1 |
| Grade 3 | 0 | 0 | 0 | 0 | 1 |
| Vomiting | 3 | 0.5 | 8 | 1.33 | 0.2244 |
| Grade 1 | 2 | 0.33 | 6 | 1 | 0.2874 |
| Grade 2 | 1 | 0.17 | 1 | 0.17 | 1 |
| Grade 3 | 0 | 0 | 1 | 0.17 | 1 |
| Myalgia (muscle pain) | 12 | 2 | 10 | 1.67 | 0.6746 |
| Grade 1 | 11 | 1.84 | 8 | 1.33 | 0.4998 |
| Grade 2 | 1 | 0.17 | 2 | 0.33 | 1 |
| Grade 3 | 0 | 0 | 0 | 0 | 1 |
| Arthralgia (joint pain) | 10 | 1.67 | 4 | 0.67 | 0.1162 |
| Grade 1 | 9 | 1.5 | 4 | 0.67 | 0.177 |
| Grade 2 | 1 | 0.17 | 0 | 0 | 0.4996 |
| Grade 3 | 0 | 0 | 0 | 0 | 1 |
| Hypersensitivity reaction | 2 | 0.33 | 3 | 0.5 | 1 |
| Grade 1 | 1 | 0.17 | 1 | 0.17 | 1 |
| Grade 2 | 1 | 0.17 | 2 | 0.33 | 1 |
| Grade 3 | 0 | 0 | 0 | 0 | 1 |
| Other | 4 | 0.67 | 4 | 0.67 | 1 |
| Grade 1 | 1 | 0.17 | 2 | 0.33 | 1 |
| Grade 2 | 3 | 0.5 | 2 | 0.33 | 1 |
| Grade 3 | 0 | 0 | 0 | 0 | 1 |
| **Total** | 272 | 45.41 | 255 | 42.5 | 0.3228 |
| Grade 1 | 266 | 44.41 | 247 | 41.17 | 0.2677 |
| Grade 2 | 33 | 5.51 | 40 | 6.67 | 0.4689 |
| Grade 3 | 5 | 0.83 | 7 | 1.17 | 0.7733 |

Data are n and proportion of participants (%). Adverse reactions and reactions were graded according to the scale issued by the China State Food and Drug Administration. Grade 1 is mild, grade 2 is moderate, and grade 3 is severe.

| **FAS** | | | | | | | | | | | | | | | |
| --- | --- | --- | --- | --- | --- | --- | --- | --- | --- | --- | --- | --- | --- | --- | --- |
| Antibody GMC | 0 | 0.5 | 1 | 2 | 4 | 8 | 16 | 32 | 64 | 128 | 256 | 512 | 1024 | 2048 | 4096 |
| D0-Zagreb group | 100 | 7.68 | 4.84 | 2.84 | 0.5 | 0.17 | 0 | 0 | 0 | 0 | 0 | 0 | 0 | 0 | 0 |
| D0-Essen group | 100 | 9.17 | 5.83 | 2.83 | 0.17 | 0 | 0 | 0 | 0 | 0 | 0 | 0 | 0 | 0 | 0 |
| D14-Zagreb group | 100 | 100 | 100 | 100 | 100 | 99.32 | 97.13 | 82.77 | 56.93 | 18.92 | 5.07 | 1.86 | 0.17 | 0 | 0 |
| D14-Essen group | 100 | 100 | 100 | 100 | 100 | 100 | 98.97 | 84.88 | 59.97 | 19.07 | 6.36 | 1.03 | 0 | 0 | 0 |
| D35-Zagreb group | 100 | 100 | 100 | 100 | 100 | 98.64 | 87.59 | 60.37 | 26.7 | 6.12 | 2.38 | 1.36 | 0.34 | 0 | 0 |
| D42-Essen group | 100 | 100 | 100 | 100 | 100 | 98.79 | 86.66 | 56.33 | 24.44 | 6.59 | 2.08 | 0.87 | 0 | 0 | 0 |
|  |  |  |  |  |  |  |  |  |  |  |  |  |  |  |  |
| **PPS** | | | | | | | | | | | | | | | |
| Antibody GMC | 0 | 0.5 | 1 | 2 | 4 | 8 | 16 | 32 | 64 | 128 | 256 | 512 | 1024 | 2048 | 4096 |
| D0-Zagreb group | 100 | 0 | 0 | 0 | 0 | 0 | 0 | 0 | 0 | 0 | 0 | 0 | 0 | 0 | 0 |
| D0-Essen group | 100 | 0 | 0 | 0 | 0 | 0 | 0 | 0 | 0 | 0 | 0 | 0 | 0 | 0 | 0 |
| D14-Zagreb group | 100 | 100 | 100 | 100 | 100 | 99.23 | 96.92 | 81.7 | 53.95 | 16.96 | 3.85 | 0.96 | 0 | 0 | 0 |
| D14-Essen group | 100 | 100 | 100 | 100 | 100 | 100 | 99.19 | 83.67 | 58.87 | 16.53 | 4.64 | 0.81 | 0 | 0 | 0 |
| D35-Zagreb group | 100 | 100 | 100 | 100 | 100 | 98.46 | 87.28 | 57.42 | 22.74 | 4.82 | 1.54 | 0.58 | 0.19 | 0 | 0 |
| D42-Essen group | 100 | 100 | 100 | 100 | 100 | 98.99 | 85.69 | 53.83 | 21.77 | 4.44 | 1.41 | 0.81 | 0 | 0 | 0 |

Table S2: Pre- and post-immunization reverse cumulative distribution in different vaccination groups after rabies vaccination. (FAS/PPS)

Data are the cumulative frequency distribution of antibody concentrations for the Zagreb group and the Essen group 14 days after the first dose and 14 days after the complete vaccination.
